# Periodic DFT Calculations—Review of Applications in the Pharmaceutical Sciences

**DOI:** 10.3390/pharmaceutics12050415

**Published:** 2020-05-01

**Authors:** Anna Helena Mazurek, Łukasz Szeleszczuk, Dariusz Maciej Pisklak

**Affiliations:** Chair and Department of Physical Pharmacy and Bioanalysis, Department of Physical Chemistry, Faculty of Pharmacy, Medical University of Warsaw, Banacha 1 str., 02-093 Warsaw, Poland; annamazurek21@gmail.com (A.H.M.); dpisklak@wum.edu.pl (D.M.P.)

**Keywords:** DFT, periodic, crystal, API, CASTEP

## Abstract

In the introduction to this review the complex chemistry of solid-state pharmaceutical compounds is summarized. It is also explained why the density functional theory (DFT) periodic calculations became recently so popular in studying the solid APIs (active pharmaceutical ingredients). Further, the most popular programs enabling DFT periodic calculations are presented and compared. Subsequently, on the large number of examples, the applications of such calculations in pharmaceutical sciences are discussed. The mentioned topics include, among others, validation of the experimentally obtained crystal structures and crystal structure prediction, insight into crystallization and solvation processes, development of new polymorph synthesis ways, and formulation techniques as well as application of the periodic DFT calculations in the drug analysis.

## 1. Introduction—The Aim of This Review

Looking at the huge development of computational power and increasing accessibility of high-performance computing systems it is naïve to believe that any of the life sciences areas may progress or even survive without application of computational modeling. Moreover, in one of the most dynamic and interdisciplinary fields of science, that is pharmacy, which integrates fundamental principles of physical and organic chemistry, physics, engineering, biochemistry, biology, and medicine, the application of computational methods is necessary, comprehensive, and widespread. However, the interdisciplinarity of pharmaceutical sciences and wide application of computational methods in them, manifest themselves in progressing narrow specialization, even among scientists using molecular modeling in their everyday work. Therefore, an aim of this review was to gather and present the recent advances and applications in a very specific branch of molecular modeling methods, that is the DFT (density functional theory) periodic calculations applied in the field of pharmaceutical sciences.

Due to the extensive application of various molecular modeling methods in pharmacy it would be impossible to even briefly summarize them in this work. The number of books on this topic is large and increasing, together with the number of more general review papers [1,2,3,4,5]. Therefore, in this short introduction to the main part of this review only two aspects will be discussed, which can be briefly summarized in two questions: “why periodic?” and “why DFT?”. Additionally, the intention of the authors is not to go into the details describing periodic DFT methodology as there is a huge number of excellent books and review articles on this topic [6,7,8,9,10] but rather to introduce the scientists working in the pharmaceutical sciences area that are not familiar with this specific type of calculations with the most recent advances in this particular field. Our motivation behind writing this review was to present the usefulness and versatile application of the presented computational methods, together with the selected case studies and in that way inspire more researchers to use such calculations in their studies, as well as to explore new, so far undiscovered applications.

### 1.1. Why Periodic? Importance of Solid State in Pharmacy

It is not without reason that approximately 70% of the drug products marketed worldwide are formulated in oral solid dosage forms [11]. Among other advantages, that type of unit doses are convenient to use, economical in manufacturing, most stable and most convenient in packaging, shipping and transportation. Simultaneously, the chemistry of solid-state pharmaceutics is characterized by high complexity and includes aspects such as, among other, crystal growth mechanisms, phase equilibria and transformation mechanisms, polymorphism, reactions at surfaces, defect interactions analysis, or structural studies.

The pharmaceutical solids can be either amorphous, exhibiting only close-range order, or characterized by molecular arrangement displaying long-range order in all directions (crystalline) or in one or two directions (liquid crystals) [12]. Further, solid drugs can be classified as single or multicomponent compounds such as crystalline solvates (including solid hydrates), cocrystals, and salts [13]. Different crystalline structures of one substance are called polymorphs [14]. The type of polymorphism that exists as a result of different crystal packing of rigid molecules is called a packing polymorphism. A more common phenomenon in pharmaceutical solids is conformational polymorphism, typical for flexible molecules and resulting from crystallization of different conformers [15]. At specified environmental conditions, such as temperature, humidity, and pressure, only one polymorphic form is thermodynamically stable and possess the lowest free energy, while all other forms are metastable or unstable [16].

Variations of pharmaceutical solid forms can result in alternations of physicochemical properties of a drug product, which as a consequence may affect drug effectiveness, safety, and processing [17]. For example, the differences in solubility observed between crystalline and amorphous active pharmaceutical ingredients (API) may be as high as several hundred times [18]. Some solid hydrates are characterized by even ten times lower solubility when compared to their anhydrous forms [19], whereas dissolution rate is usually increased significantly in salts and cocrystals [20] when compared to the single component compounds. Solid form structure affects crystal morphology, which may have an impact on processability of the drug product due to the different mechanical and flow properties [21]. For example, needle shaped crystals are usually undesirable for pharmaceutical applications since they are difficult to handle in the formulation process [22]. Additionally, the crystal morphology greatly influences the bioavailability of the inhaled drugs, i.e., bronchodilators [23].

During the drug analysis process, as a result of dissolution of APIs or solid formulations, some chemical information, i.e., on the polymorphic form or solid-state interactions, are irrevocably lost. Therefore, to overcome this problem solid state methods of analysis found their wide applications in pharmaceutical sciences [24]. For example, solid state nuclear magnetic resonance can be used as a perfect tool to study the pharmaceutical solids, both APIs and final dosage forms [25]. However, both the solid-state analysis itself as well as the interpretation of its results is in many cases much more complex than in the case of liquid state samples [26].

As stated above, the physical and chemical properties of solid-state APIs, resulting from the arrangement of molecules in the solid state, are related to their stability, solubility, bioavailability, and formulatability. Therefore, the possibility to accurately predict and describe those properties using molecular modeling methods is both interesting from the purely scientific point of view but also of a great practical importance in the pharmaceutical industry. As will be later described, molecular modeling methods in the case of solid APIs are usually used to predict their physicochemical and structural properties, to explain the experimentally obtained results, or to predict the conditions required to obtain the new forms of solid pharmaceutics in order to minimize the number of experiments or optimize the experimental conditions. Further, the calculated properties such as Nuclear Magnetic Resonance (NMR) shielding constants or Raman/Infra Red (IR) frequencies may greatly facilitate the solid-state analysis.

However, since the unique for each solid form features arise from the short and long-distance intermolecular interactions, molecular modeling methods in which the single molecule in vacuum or in solution is being modeled were found to be inappropriate and inaccurate. While those “single molecules” methods are generally successfully applied in other aspects of pharmaceutical sciences, i.e., to study the drug-biomolecule interactions or to predict the formation of complexes, to study the solid state pharmaceutics other type of calculations should be applied, which will be described in the next section.

### 1.2. Why Density Functional Theory (DFT)? The Differences Between the Isolated Molecule and Solid State Modeling

There are many ways to classify the molecular modeling methods, however they are usually divided based on their atomistic level description of the molecular systems. This may include treating atoms or even whole functional groups as the smallest individual rigid units (a molecular mechanics “MM” approach), or explicitly modeling nuclei and electrons (a quantum chemistry “QC” approach). Without going into details, the main practical differences between those two types of calculations are the speed of computations and accuracy of the obtained results. While MM methods are always faster, they do not provide the reliability and accuracy of high-quality QC calculations. Therefore, the choice of the molecular modeling method depends on the size of the studied system and the available computational power as well as the time intended for calculations. Nowadays, both commercial and license free software that enable the periodic calculations on molecular systems are available. The list of the most popular programs together with their selected properties is presented in Table 1.

As mentioned before, MM methods are widely used in pharmaceutical sciences i.e., to calculate binding constants [36], study protein folding kinetics and mechanics [37], or complexation equilibria [38]. However, in the field of solid pharmaceutics, the energy differences between experimentally obtained polymorphs are usually less than 1 kcal/mol but sometimes can even be lower than 1 kJ/mol [39]. Therefore, to ensure an accurate stability ranking of low energy polymorphs, calculations with sub-kJ/mol accuracy are necessary [40]. Additionally, the structure of solid pharmaceutics is stabilized by multiple types of specific intermolecular interactions such as hydrogen bonds, π-π stacking, C–H-π interactions, halogen bonds, dipole–dipole interactions, or van der Waals interactions. Unfortunately, to describe all these interactions correctly, MM force fields can be insufficient and thus the use of QC methods is usually required. In some cases, to improve the accuracy of the results without the extensive elongation of their time the combination of MM and QC methods is applied, i.e., MM methods are used for screening and initial calculations while QC methods are adapted to improve the final results. Such a hybrid approach was found to be useful i.e., in some of the crystal structure prediction (CSP) studies [41].

The opposite of MM methods, in terms of both accuracy and speed of calculations, are post Hartree–Fock calculations that have recently became possible for organic solids [42]. For example, periodic local second-order Møller–Plesset perturbation theory (MP2) has been successfully applied to several small molecular crystals [43]. Another very promising high-level first-principles method is diffusion quantum Monte Carlo [44], however as in any of the QC methods, the required computation time scales rapidly with system size, which makes this option unavailable for most researchers. One of the most popular molecular modeling methods that has emerged in the last few years, combining high accuracy and reasonable computational cost, is the application of solid state periodic DFT calculations.

DFT has been very popular for calculations in solid-state physics since the 1970s. However, the rapid development and application of this method in modeling molecular crystals started in the 1990s [45]. Below, some important aspects of DFT periodic calculations will be briefly summarized.

#### 1.2.1. Periodic Boundary Conditions

For accurate and computationally feasible approximation of a large system such as macroscopic crystals, periodic boundary conditions are often applied using crystal unit cells as simulation boxes. During the computations only the properties of the original unit cell need to be calculated and then propagated in the chosen dimensions.

One of the features that differentiates the software capable of periodic DFT calculations is the type of system that can be modeled. Most of the programs are specifically designed for the three-dimensional periodic systems such as crystals. However, there are some codes that enable calculations on any type of system, such as three-dimensional crystals, two dimensional slabs, or one dimensional rods and even single molecules without applied periodicity (Table 1). This is particularly helpful, for example, when calculating the cohesive energy or phenomena such as crystallization or sublimation. However, even using “only 3D” software the calculations on single molecule, simulating gas phase, can be performed by creating a sufficiently large empty cell and putting the studied molecule inside it. This approach is usually referred to as a “molecules in the box” type of calculation.

Additionally, the main advantage of imposing periodic boundary conditions relates to Bloch’s theorem, which states that in a periodic system each electronic wavefunction can be written as a product of a cell-periodic part and a wavelike part. The cell periodic part can then be expanded using a basis set consisting of a discrete set of plane waves whose wave vectors are reciprocal lattice vectors of the crystal. Therefore, each electronic function can be written as a sum of plane waves [46].

#### 1.2.2. Pseudopotentials

In the DFT approach, the electronic structure is evaluated on the basis of a potential acting on the electrons in the system. The DFT potential is constructed as the sum of external potentials, determined solely by the structure of the system and an effective potential resulting from interelectronic interactions. All-electron DFT methods treat core and valence electrons in the same way. However, the DFT calculations can be very much simplified and accelerated if electrons are divided in two groups: valence electrons and inner core electrons. In most cases, the electrons of the inner shells (core electrons) are tightly bound and are not involved in the chemical binding. In most organic molecules, binding is solely due to the valence electrons [47]. This separation means that in a large number of cases the atom can be reduced to an ionic core that interacts with the valence electrons. In the widely used pseudopotential approach, ion cores are considered to be frozen, meaning that the properties of solids are calculated on the assumption that the ion cores are not involved in chemical bonding and therefore they do not change as a result of structural modifications or presence of other atoms. A pseudopotential represents an effective interaction that approximates the potential felt by the valence electrons [48]. Alternatively, some of the programs used for periodic DFT calculations, i.e., DMol3, enable the user to choose between the all-electron and pseudopotential approach.

#### 1.2.3. Plane-Wave Basis Sets

Besides the localized basis sets, commonly applied in the non-periodic DFT calculations, plane-wave basis sets can also be used in QC computations. These basis sets are very popular in calculations involving three-dimensional periodic boundary conditions. The main advantage of a plane-wave basis is that it is guaranteed to converge in a smooth, monotonic manner to the target wavefunction [49]. In contrast, when localized basis sets are used, monotonic convergence to the basis set limit may be difficult due to problems with over-completeness: in a large basis set [50]. Additional benefit resulting from the application of plane-wave basis set is the introduction of periodic conditions to the studied system.

#### 1.2.4. Symmetry of the Studied System

The application of symmetry operators is crucial for achieving high performance of plane-wave basis sets calculations. Usually a system characterized by a high symmetry has a relatively small unit cell which is reflected by the large number of k-points required for efficient Brillouin zone sampling. This number can be greatly reduced if it is recognized that the contributions to the charge density from wavefunctions with k-vectors that are related by symmetry. Because the computational cost of a periodic DFT calculations using plane-wave basis set increase linearly with the number of k-points, any symmetry-related reduction in the number of k-points will give a direct computational speedup.

#### 1.2.5. Dispersion Forces

For a long time, the main weakness of DFT calculations was their inability to account the dispersion interactions. While this was not a major problem in the case of systems characterized by strong electrostatic interactions such as ionic solids, it was a serious limitation for molecular crystals, where dispersion forces such as van der Waals interactions greatly contribute to the overall binding energy. The most popular method to overcome this problem is the application of “dispersion corrections” (DFT-D) of the form C_6_R^−6^ in the DFT formalism [51]. These semiempirical approaches provide the best compromise between the cost of first principles evaluation of the dispersion terms and the need to improve non-bonding interactions in the standard DFT description [52]. The alternative way to consider the dispersion interaction is by using the nonlocal van der Waals (NL-vdW) functionals [53], that are being applied more and more frequently in solid-state calculations, as they have shown to be much more reliable than the traditional semilocal functionals. In a recent study [54], an assessment of NL-vdW functionals was presented. A dozen of dispersion-corrected functionals have been tested on periodic solids with the goal to identify which of them are the most appropriate. The authors suggested that among the tested functionals, rev-vdW-DF2 [55] was found to be very accurate both for weakly and strongly bound solids.

## 2. Application Areas of Periodic DFT Calculations in Pharmaceutical Sciences

### 2.1. Validation of the Experimentally Obtained Crystal Structures and Crystal Structure Prediction

One of the earliest ideas to use the periodic DFT calculations in pharmaceutical sciences was to apply them in order to check whether it is possible to theoretically reproduce and compare the experimental and computational results, starting from the crystal structure verification and other types of structural studies [56]. Nowadays, it is clear that such an approach usually ends up with reasonable, repeatable results [57,58,59,60].

#### 2.1.1. Powder X-Ray Diffraction (PXRD)-Based Structure Prediction and Verification

DFT periodic calculations have been already reported many times as a useful method to support the discovery and characterization of a new polymorph [61,62]. Usually, the experimentally obtained crystal structure is subjected to DFT geometry optimization which allows the comparison of the initial (experimental) and calculated structure. In most cases, especially when the experimental structure originated from the single crystal X-ray diffraction (SCXRD) analysis, the theoretical results are found to be in excellent agreement with the corresponding experimental ones [63,64]. However, usage of such type of calculations in this field is definitely not restricted to the evaluation role. The usefulness and complementarity of DFT periodic calculations is particularly visible when combined with powder X-ray diffraction (PXRD) analysis.

In most situations the experimental PXRD patterns are used to access some information about crystal structure when SCXRD analysis is unavailable [65]. It is a common situation that newly obtained API is crystalline, which can easily be proven by the PXRD analysis, however the quality of experimentally obtained crystals is not sufficient for the SCXRD measurements. PXRD is also of a great use in situations when a single crystal could never be obtained, as it happens when some polymorphs can be reached only after spray-drying or milling [66]. Crystal structure determination using powder diffraction is difficult and may be inaccurate [67]. Often, in the PXRD, experimentally obtained crystal structures of organic solids the positions of the hydrogen atoms are not included and a DFT geometry optimization before further calculations is necessary [68]. However, it is also possible, without applying any DFT calculations, to generate PXRD pattern of the crystal structure obtained from DFT calculations, what facilitates understanding of the molecular interactions in a crystal, as in case of a praziquantel and calcium carbonate [69]. As from the PXRD pattern analysis the unit cell dimensions and space group can be relatively easily determined by using, for example, Pawley refinement, such information can serve as a quick and direct validation of the theoretical calculations as well as the data that can be used to construct the initial unit cell for crystal structure predictions studies, described in the next section.

**Figure 1:**
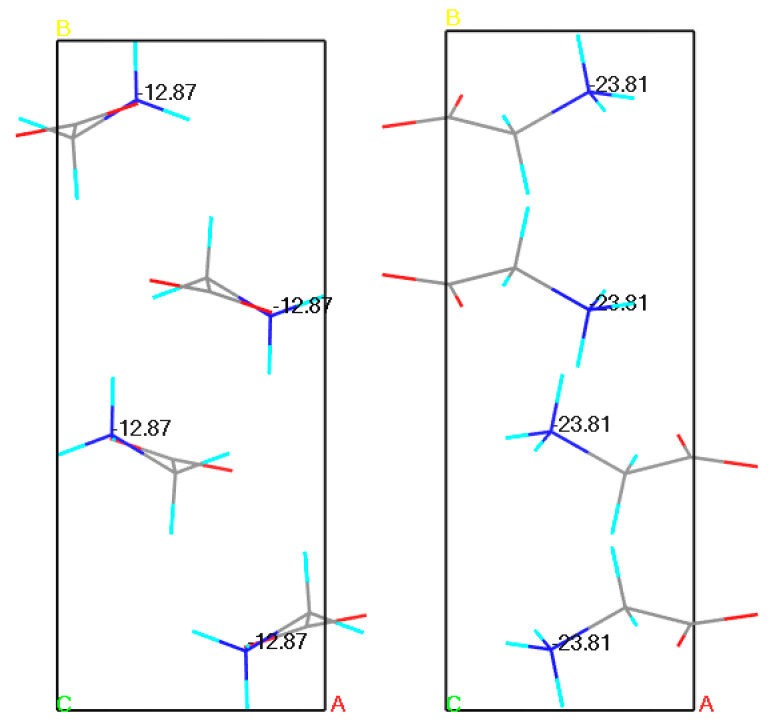
Results of Gauge Including Projector Augmented Waves Nuclear Magnetic Resonance (GIPAW NMR) chemical shielding anisotropy calculations for the N atom of the α glycine crystal structures. On the left powder X-ray diffraction (PXRD) structure (−12.87 ppm), on the right single crystal X-ray diffraction (SCXRD) structure (−23.81 ppm). Experimental value: −12.35 ppm. Source: author’s archive, more details [71].

It has also been reported that when the inaccurate, yet experimentally obtained crystal structure, is used for the calculations, the results may be incorrect, accordingly with the garbage in–garbage out concept [70]. For example, in one of our earlier studies we have performed a series of similar NMR parameters calculations for α glycine using all available crystal structures from Cambridge Crystallographic Data Centre (CCDC). In this large group (39) of crystal structures, calculated NMR parameters using low quality structure obtained from the PXRD differed significantly from the ones obtained using structures originating from SCXRD experiments, even after performing geometry optimization (Figure 1) [71].

**Figure 2:**
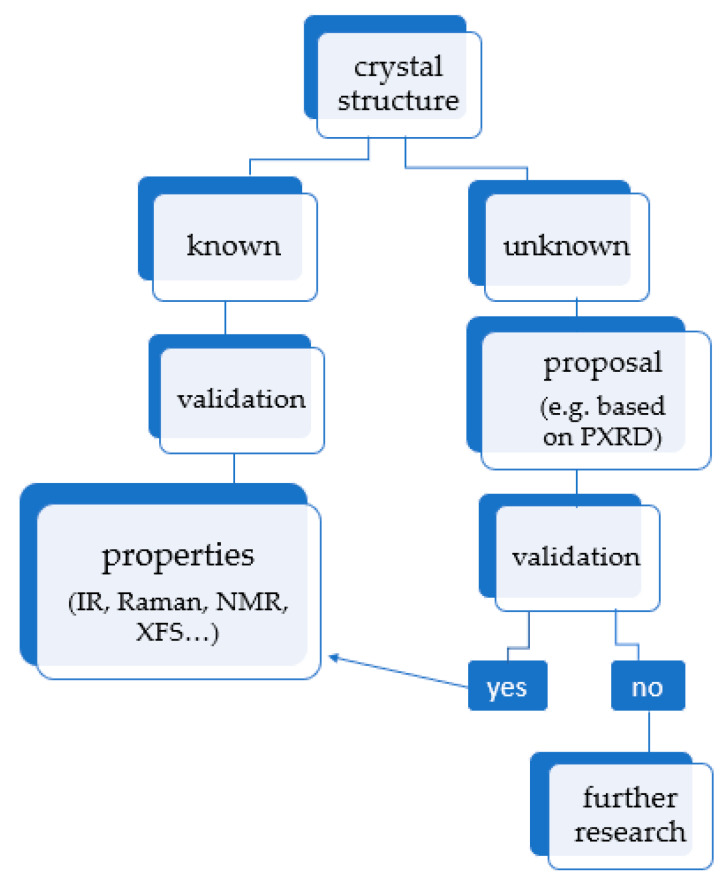
Possible options in the area of periodic density functional theory (DFT) calculations for crystals. Once the crystal structure is obtained and validated, it can be used to calculate other properties, such as thermodynamics or spectroscopic data. XFS stands for X-ray fluorescence.

Encouraged by the success of those initial computational experiments, such calculations started to be applied in order to predict or solve the crystal structure rather than confirm or correct it (Figure 2).

#### 2.1.2. Crystal Structure Prediction

PXRD analysis-resulting data can be also an element of a much wider concept, called Crystal Structure Prediction (CSP) [72]. Most commonly it is used either to solve the new crystal structure when some experimental data is available in order to support the theoretical assumptions or to predict the new polymorphic form and the conditions that are necessary to obtain it [73,74,75].

It has been already shown that such combination of theoretical and experimental data can be a starting point for determination of a crystal structure. The evidence provides the case of axitinib [76]. The applied CSP methodology enabled an accurate identification of four previously known axitinib polymorphs. Worth mentioning is also a relatively recent case study of selexipang form I [77]. Most probably, it is one of the most complex structures solved ab initio using solely DFT calculations and the powder diffraction data.

An even more striking example is the way in which the fifth polymorph of dapsone has been found and described [78]. Previously, the kinetically stable form III has been known. However, the implementation of the DFT-based CSP methodology into the research enabled finding new, this time metastable, form V. The crystal structure has been solved and described as one with untypically high number of molecules in the asymmetric unit (Z’), Z’ = 4. In this case, due to the usage of DFT calculations, not only a proper (proven by calorimetry measurements) thermostability order of polymorphs has been obtained but also an explanation for the high Z’ has been given, namely dense packing and more stable intermolecular interactions in form V than in form III.

In the topic of de novo construction of a crystal, a case of benzoic acid-based molecule should be noted [79]. The aim of this study was to produce theoretically as many reasonable possible structures of the investigated compound as possible and after computational selection among them, to prove the accuracy of the choice. Periodic DFT calculations helped to optimize the structures and were applied for NMR data calculations. As a result, a previously unknown crystal structure has been successfully determined.

CSP is not only an attempt to find new polymorphic forms but also to use the periodic DFT calculations in order to revise the data which has been already achieved experimentally. Sometimes, after application of the periodic DFT calculations, a question emerges whether the old, dating back to the 1970s available in CSD data, should be redefined. A report on cefradine dihydrate and cefaclor dihydrate serves as an example [80]. For those chemotherapeutics, the theoretically obtained structures have been subsequently confirmed by the single crystal experiments and as a result the previously not available atomic coordinates have been determined. The authors of this work claim that the structure description accuracy of modern DFT calculations is most probably higher than the one of the experiments from the 1970s. The further research in this field is still needed but this example shows the utility of periodic DFT approach to check and correct crystal structures already accepted decades ago.

Another example is 4-Aminoquinaldine monohydrate which was known to be a dense-packed crystal exhibiting strong H-bonding. Surprisingly, the calculations revealed that the considered structure (named afterwards Hy1A) is not a global energy minimum on the calculated crystal energy surface [81]. The further research showed that there exists another thermodynamically stable mono-tropically related 4-aminoquinaldine monohydrate polymorph (Hy1B). But a phase transformation Hy1A → Hy1B takes place only in case an impurity is present. This study shows the potential of DFT calculations to predict possible dangers while crystallization process. It could be a useful tool for the industry and production of pharmaceuticals as well.

A similar example is a transformation of Etoricoxib form I into form IV [82]. The transition happens at the elevated temperature but solely when a sample already contains a small amount of the form IV. Such a mechanism was supported by the total energy calculations. This plainly indicates that the theoretical approach can effectively play a complementing role to the experimental research and suggests that it is even capable to predict the atypical routes of crystallization. What is more, it can also reveal the potential usefulness of some specific substituents in already known substances [83] and therefore speeds up the laboratory synthesis. Moreover, it helps to state sometimes quite unusual hypothesis, which probably would not be revealed otherwise, like the existence of a new pyrazinium hydrogen sulfate P1 polymorph with an unusually high dipole moment [84].

Different approach is the usage of calculations to find an isostructural template facilitating crystallization [85]. Such computationally-guided isomorphus template induced crystallography and revealed a completely new metastable polymorph of cyheptamide [86].

### 2.2. Insight Into Crystallization and Solvation Processes

It has been proven that the discussed theoretical methodology helps to understand the mechanism of crystallization and has been already successfully employed for the description of, among others, co-crystallization.

#### 2.2.1. Co-Crystals

*N*-Salicylideneaniline molecular switch is characterized by thermochromism and depending on the intermolecular interactions with the co-former, takes either enol or keto form [87]. In the recent study seven different co-formers, interacting with the investigated molecule via H- or halogen- bonding, have been studied. It was an application of periodic DFT calculations which allowed to describe the enol-keto relative energy, geometry of the co-crystals and electron distribution within the structures. It has been stated that in 6 out of 7 cases, enol form dominates. Such conclusion stays in agreement with the experiment. The application of theoretical calculations enabled description of the relative stabilization of the systems obtained upon co-crystallization. It also supported the knowledge on the reduction of thermochromism of the investigated *N*-Salicylideneaniline molecular switch, which takes place after co-crystallization with several co-formers.

Theoretical investigation can be also applied to understand the sole role of different co-crystallizers. This happened in case of N4-acetylsulfamerazine when it was revealed that acetic acid destabilizes the structure of API and forms the least stable, yet the best soluble of all takes into account co-crystalline systems [88]. Moreover, this type of research enables to point out what kind of thermodynamics factors have a dominant influence on the mechanism. In the case of N4-acetylsulfamerazine, the entropic effect has been assigned as the one which determines the process of the nucleation.

The basics of co-crystallization mechanism refers also to a case of benzoic acid and sodium benzoate: a rare case of a co-crystal polymorphism [89]. This fact explains why inner structures and such structural composition underlying factors have been deeply investigated. Theoretical approach enabled determination of a thermodynamic relationship between two co-crystals, which turned out to be of an enantiotropic character. It has been stated that in that case the packing polymorphism dominates. However, the conformational polymorphism is said to be partly responsible for the final structure, as well.

The case of droperidol and benperidol can serve as another good example [90]. Although these two antipsychotic drugs form isostructural phases, they do crystallize in different solvates. On the basis of the lattice energies of polymorphs as well as hydrates, it has been proven that the structural differences between two substances do not contribute significantly to the sum of energy from all intermolecular interactions. Thereby, there must exist another reason for the observed phenomenon. The explanation are intermolecular interactions which are energetically more favorable in droperidol. As a result, only droperidol forms channel solvates, whereas for benperidol a different inner structure is obtained. Such a conclusion could have been made only thanks to implementation of DFT calculations into the interpretation of PXRD- and Thermal Gravimetric Analysis (TGA)-derived data.

##### 2.2.2. (De)Solvation and Dehydration

The above described example introduces the next important topic: dehydration. This process is especially interesting to be investigated, as it can proceed in various ways and ends up with a collapse of a crystal lattice, reorganization of the whole crystal structure, or can lead to only slight changes in the initial structure [91]. What is more, it can be either continuous or progress in steps.

To study the chemistry of solid organic solvates, one of the most common approaches is to describe the hydrogen bonds network which arises between water molecules and investigated structure, and in the second step, to compare this net with the system generated without a solvent [92]. The second attitude to this topic is a step-by-step depiction of dehydration through investigation of consecutive hemihydrates [93]. So far, for theoretical calculations, the hemihydrates must be created manually by systematically removing water molecules from the system. However, the possibility of studying the hemihydrates is also available via the supercell approach described in Section 1.

The subject of dehydration is of great importance for the industry and it often relies on the relative humidity and phase purity [94]. Hence, the understanding of the mechanism behind dehydration is of high value for the controlling product quality throughout its processing. In many cases, computationally determined dehydration enthalpy agrees with the experimental one. From a purely scientific point of view, it is also interesting when dehydration as well as desolvation of the same substance can be measured/calculated and compared. This happens in the case of orotic acid [95]. Its dimethyl sulfoxide mono-solvate and its monohydrate, when the proper desolvation conditions are provided, change into the same anhydrous form. This mechanism can be explained within the DFT calculations which shed new light on the moisture-dependent stability of the investigated substance.

An even more complex description of a solvent-induced phase transition is available for acyclovir (ACV) which forms ACV: H_2_O 3:2 hydrate (form V), ACV: H_2_O 1:2 dihydrate (form VI), and four different anhydrous forms (form I, II, III, IV) achieved upon up-heating, moisture change or dissolution in methanol [96]. Periodic DFT application significantly contributed to the full understanding of the reasons for solvates stability and structural changes upon desolvation or modification of physical parameters. It has been described in detail that the water molecules in form VI are located in the wide channels and form a dense H-bonding network. Such packing explains rapid temperature-induced transformation into more stable form V. While the V → I process could be structurally easily understood as in both these systems ACV molecules are paired in similar dimers, the transformation V → II is of a much more complex nature due to the significant differences in the inner structures. This explains why form II can be obtained from form V only in a solvent-mediated experiment. Additively to the pure geometry optimization of the ACV forms, in order to support structural information, the DFT-NMR shielding calculations have been performed.

Periodic DFT calculations performed on the solvates provide both a thermodynamic stability order [97] and a deeper understanding of the experimentally obtained data. This means that a complete description is available, including the explanation for the stability of a form owning some specific characteristics. One of the examples could be relatively high Z’ (as it is in case of phenantroline monohydrate, where Z’ = 3 [98]). Theoretical approach explains that the interplay between the structure stability and its high Z’ has source in the density of a crystal packing. What is more, this type of calculation plays an important role in the overall investigation of new polymorphs, especially as many of them are awaited to show enhanced solubility [99,100,101]. Here, an accurate example is a research conducted on co-crystals of 4-hydroxybenzamide (4-OH BZA) with salicylates: salicylic acid, 4-aminosalicylic acid (PASA), acetylsalicylic acid, and salicylsalicylic acid [102]. To describe the non-covalent interactions in the co-crystals, the DFT calculations were used. The most promising results have been obtained for PASA. A combination of experimental and theoretical data points out the better solubility of the PASA-4-OH BZA, when compared to pure API. Moreover, the co-crystal exhibits higher stability what prevents irreversible decarboxylation of PASA to the toxic 3-aminophenol. Although such a deduction could not be based solely on the DFT calculations, the implementation of the theoretical approach helped to depict the inner structure of newly grown co-crystals and to complement the knowledge on their properties.

In some cases, the structural similarity between an original substance and its solvate, as well as thermodynamic stability of the emerging solvate polymorphs must be taken into account. This usually complicates the description of the process and the accurate determination of the stability as there emerges need to consider both pairwise intermolecular interaction energies and the lattice energies [103]. Nevertheless, even then, periodic DFT calculation is a useful method to evaluate the stability order and gives a very quick insight into the mechanism of the solvation process.

### 2.3. Development of New Polymorph Synthesis Ways and Formulation Techniques

The usefulness of periodic DFT calculations goes beyond solely the discovery of a new polymorphic forms. One of their application areas is the surface modelling. The research in this topic refers to various surface utilization possibilities in drug development-related sciences, as drug’s adsorption and release from a drug delivery medium [104,105,106], tableting process (slip agents, optimization of tableting process parameters) [107], elucidation of contaminants, or surface-induced polymorphism [108].

In one of the studies, carbamazepine molecule was placed on a gold surface and a periodic cell has been created [109]. The results of calculation point out to the potential of metal or templating agents in controlling the polymorph formation. A similar experiment has been reported with TiO_2_ anatase as a surface for peptide-bond formation between two glycine molecules [110]. As this reaction is crucial for synthesis of amides, the development of the study could deliver interesting data for the pharmaceutical industry.

Another very practical and current issue which has been brought up by the researchers applying the DFT periodic calculations is removal of the benzodiazepine rests. This is a rising problem for the environment and as a consequence for every human. This is why the adsorption of alprazolam and diazepam on vermiculates surfaces has been put under investigation [111]. The theoretical characterization of strong water and cation bridges with the pharmaceuticals via exchange of the Mg^2+^ ion indicates that this surface has a high potential to bind and therefore removes the rest of the benzodiazepines from the waste. A similar example is an attempt to remove the chlorpheniramine contamination [112]. In this case an energy of binding between the birnessite surface and a drug has been assessed.

Research on surface-binding is vividly present among DFT-calculations. A desire to overcome the hypersensitivity against metals in stents led researchers to a project whose aim was to replace the currently used nickel-based stent coatings with the nano-structured layers which could, at the same time, serve as a drug-reservoir [113]. The investigated TiO_2_ surface has been theoretically constructed as two-TiO_2_-layer in a supercell with a 15 Å vacuum gap.

The next system worth mentioning described with periodic DFT is an adsorption model of ibuprofen and aspirin on the amorphous silica surface [114]. A different Si-OH groups’ density determined different hydrophobic/hydrophilic character of a surface. The research confirms that the dispersion correction is necessary to properly model the investigated interactions. This is of great importance as such interactions evoke high interest in the industry as adsorption on amorphous silica is widely adopted in pharmaceutical formulations: tableting process, drug delivery systems, and anti-caking agents.

**Figure 3:**
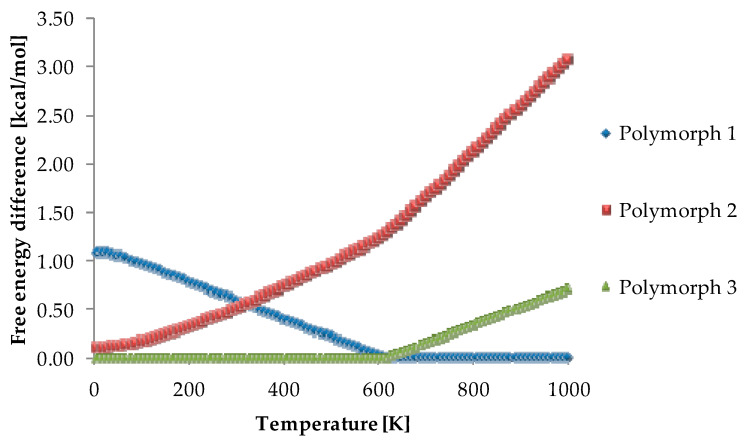
Results of thermodynamics calculations for three polymorphs of nootropic drug, piracetam. The energy of the lowest energy form at given T is, at that T, the reference one and its value is set to zero. Using periodic DFT calculations it was possible to determine the order of stability of those three studied forms. The calculated results were in agreement with the experimental data, that is the Polymorph 3 is the most stable one at low temperatures until it transforms into Polymorph 1 which is the most stable one at high temperatures. The Polymorph 2 is metastable in the whole temperature range. Source: author’s archive, more details [116].

Another topic connected with a surface property is fracture of particles. The process of micronization imposes an effect on the surface energy of a substance. An increase in polar surface can result in smearing and stickiness of the material [107]. Such changes are of a decisive importance for the drugs’ production and thanks to DFT calculations can be described and understood on the atomic level. As both temperature and pressure are imposed on the substances while the tableting process, it has in itself an immense influence on the tablet components state [115]. Under the applied conditions, a polymorphic transition may take place which can be predicted with DFT-methodology (Figure 3) [116].

### 2.4. Drug Delivery Systems

Nowadays various drug delivery systems are developed and in many aspects the research in this field can be supported by theoretical calculations [117,118]. This means from one side, the description of API, which could change its molecular structure, for example, upon uploading into drug carriers, as it takes place in case of ellipticine [119]. Thanks to ^13^C NMR measurements, it is known that this drug favors highly ordered form in contrast to its bulk initial structure. Such analysis helps to fully understand the mechanism of adsorption, as it is for halloysite nanotubes with isoniazid [120], where a strong H-bond arises between the carbonyl group, heterocyclic N atom, and the hydroxyl H atoms of the surface.

On the other hand, the interaction between drug and the delivery system can also be evaluated. Both the analysis of absorption and the vibrational frequencies have been obtained for carbon nanotubes and small molecules [121]. A layered polymer, like poly(lactic-co-glycolic acid) [122], and its affinity to a drug, could be assessed with the DFT-technique as well.

An interesting example is a complex guest-host interaction while nano-crystallization of tolbutamide form V [123]. Depending on the loading process, either highly dynamic amorphous/ disordered state or a direct crystallization into metastable form V emerges. The processes could be fully described by implementing DFT-calculations into the research.

Quite a different group of possible drug delivery systems are nanotubes. A numerous research has been already conducted on the drug and vehicle binding. A definitely predominant approach is molecular DFT-D accompanied by the Molecular Fraction with Conjugate Caps methodology [124,125,126]. However, in order to investigate the adsorption of isoniazid and pyrazinamide on the nitrogen-doped single-wall nanotubes, the periodic DFT approach has been applied [127]. Interestingly, a similar pattern of adsorption has been observed for both periodic and cluster models.

A slightly different topic, but still in nano dimension, are gold nanoclusters and nanoparticles which could be used as biosensors in medical diagnostics. Such an application motivated the investigation of intermolecular interaction between serine and threonine dipeptides and the above mentioned nano-medium [128]. The values calculated with B3LYP functional stay in good agreement with the experimental data.

### 2.5. Stability of Polymorphs

#### 2.5.1. Pressure and Temperature Stability Dependence

The already brought up amino acids are constantly undergoing detailed research, especially on the polymorphic stability, in which periodic DFT methodology is implemented. A small number of atoms enables more sophisticated calculations with relatively low computational cost. This is why thorough the analysis of proline [129], l-cysteine [130], l-alanine [131], glycine [132], l-tryptophan [133], l-serine [134] has been conducted. The research encompasses generation of the NMR spectra, vibrational analysis, assignment of signals, H-bonding arrangement.

**Figure 4:**
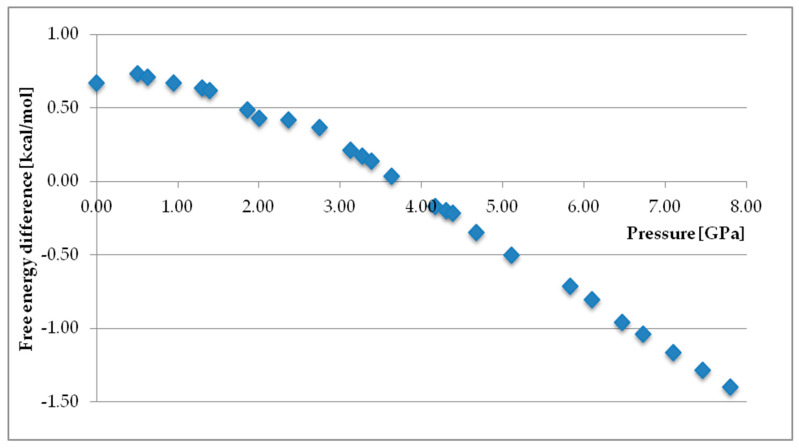
Results of thermodynamics calculations at constant temperature (298 K) and variable pressure for crystalline glycine, the differences between the free energy of δ and α polymorphs. Calculated values support the experimentally observed high pressure induced phase transition of α to δ polymorph. Source: author’s archive, more details [139].

As in previously reported cases, also here the lattice energy is calculated. However, in reality such an approach refers only to the state of T = 0 K [135,136] (and this is also only a roughly calculation as zero-point energy is not taken into account). Putting large molecules under investigation which takes into account temperature contribution, would take too much calculation time in order to obtain precise results. However, amino acids are a perfect model structure to include the temperature and pressure effects and as a result arrive at relative stability for desired thermodynamic conditions, e.g., standard temperature. An example is prediction of glycine crystal structure performed entirely from the first principles [137]. It revealed the thermostability order of glycine polymorphs, including the two high-pressure forms (Figure 4). Most importantly, such an approach helps to predict the existence of new polymorphs in experimentally not explored temperature or pressure ranges and relatively precisely determine the thermodynamic parameters in which a phase transition takes place as well as proposes an explanation for the molecular changes during the transformation. In the last years, special interest has grown in the calculation including high pressure [138,139,140].

A very good example of mutual completion of experimental and computational studies in high pressure circumstances is case of L-serine. A detailed research has been undertaken, including Raman [141], neutron powder diffraction [142], X-ray [143], and PXRD [144] analysis. Firstly, the obtained data served to perform MM calculation (PIXEL) in order to access the intramolecular interactions in the structures [145]. At this stage also QM has been implemented to check whether it is possible to theoretically reproduce the experimental parameters, which is the pressure of a phase transition. The calculated results were comparative with the known pressure values [146]. Afterwards, fully theoretical, periodic DFT study on L-serine has been reported. This in the end enabled a full understanding of the mechanism behind the investigated phase transition [147]. The modelled effect of applied pressure on the H-bonding helped to rationalize experimentally observed radical differences between polymorph changes I → II and II → III.

Another study which provides a complete and vast information on the pressure-induced polymorph transition is case of trimethylamine [148]. Not only are the obtained equilibrium lattice parameters in good agreement with the experimental data but also the calculated enthalpies do properly assess the relative stability of phases. Moreover, the existence of two additional phase transitions has been supported. All the above enumerated information has been gathered in one thoroughly theoretical study. This shows how highly developed a software can be which uses periodic DFT in high-pressure research.

An even more outstanding example is the very recent investigation of L-threonine. It combines single crystal, X-ray, Raman, neutron powder diffraction, MM, and periodic DFT measurements and reports one of the highest pressures described for a complex molecular material: 22.3 GPa with a detailed information on the system [149].

The development of periodic DFT, applied for analysis of structures emerging in high pressure, helps also to reveal and rebut some misconceptions from the past. This happened with urea molecule. For decades it has been widely accepted that a polymorph II exists. However, after its apparent discovery by Birdgman in 1916, it has been never experimentally achieved. It was only in 2019 when the research proved the nonexistence of phase II and its coincidence with phase IV in room temperature [150]. Such an experimental approach has reopened the computational investigation of urea structure as only phase I, III, and IV own a detailed structure description [151]. This example shows how theoretical calculations help to clarify the uncertainty concerning phase transitions and point out new directions of research.

However, in this topic of imposed outer parameters, periodic calculations are also implemented inversely: not as an achieving but verifying tool for new structures. This is how eventually the existence of the obtained during the direct compression, high-stability ethenzamide-aliphatic dicarboxylic acids co-crystals have been proven [152] or structural differences in the diethylcarbamazine citrate at 293 K and 235 K have been described [153].

#### 2.5.2. Molecular Dynamics

The subject of the temperature implementation leads to another calculations’ type, namely molecular dynamics (MD). The widely used Velocity Verlet scheme [154], which integrates Newton’s equation of motion, models systems with a constant temperature or pressure conditions (constant number of molecules (N), volume (V), and temperature (T)–(NVT); constant number of molecules (N), pressure (P), and temperature (T)–(NPT) ensemble). Therefore, it is possible to observe, for example, folding and unfolding of a cellulose II chain [155]. MD is often used to complement the wide-scale research of a structure [156]. It is found especially useful in QM/MM approach, e.g., to model epinephrine in water solution, where the outer solvation shells are treated with molecular mechanics [157] and MD is applied for the drug molecule and its nearest surrounding. Fifty-two uncorrelated snapshots have been chosen to simulate the electronic absorption spectra of epinephrine. The agreement between theoretical and experimental data shows that the compilation of Car-Parinello MD [158] and time-dependent density functional theory (TD-DFT) can be successfully used to obtain an electronic absorption spectrum.

MD can be also incorporated in the investigations of additives because it enables description of an adsorption process which afterwards undergoes a comparison with the experimentally achieved data. This could improve the product specification preparation and process development. For example, the mechanism of a surfactant adsorption on the nifedipine crystal has been described as one based on the physical interactions. Such a conclusion emerged because MD ruled out any chemical interaction [159].

**Figure 5:**
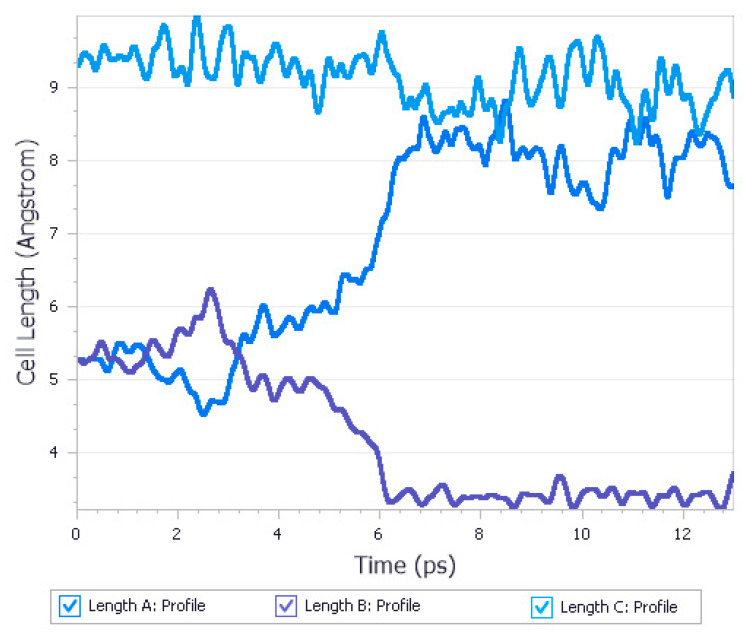
Results of molecular dynamics calculations at 3.10 GPa for urea Form I, unit cell lengths profiles. A phase transition is observed after 6 ps of simulation. The results were found to be in agreement with the experimental data as Form I is metastable and transforms into Form IV at 3.10 GPa. Source: author’s archive, more details [160].

Recently, periodic DFT MD calculations have been used in one of our studies to model the polymorphism of urea [160]. Crystalline urea undergoes polymorphic phase transition induced by the high pressure. Form I, which is the most stable one at normal conditions and Form IV, which is the most stable one at 3.10 GPa, not only crystallize in various crystal systems but also differ significantly in the unit cell dimensions. The aim of that study was to determine if it is possible to predict polymorphic phase transitions by optimizing Form I at high pressure and Form IV at low pressure. While after geometry optimization of Form IV at 0 GPa Form I was obtained, performing energy minimization of Form I at high pressure did not result in Form IV. However, employing MD dynamics calculations enabled to accurately predict this high-pressure transformation (Figure 5).

#### 2.5.3. Lattice Dynamics and Phonons Calculation

Calculated through density functional perturbation theory (DFPT), the phonon density of states enables a precise description of a thermodynamic state [161] enabling calculations of Gibbs free energy, enthalpy, entropy and zero-point energy. For example, a quite commonly performed comparison between experimental sublimation enthalpy and the calculated lattice energies requires the vibrational contribution, which is determined via phonon calculations [162,163]. As lattice vibrations contribute to the entropy [131], they also directly influence the Gibbs free energy, and as a consequence, the thermodynamic stability of a crystal.

### 2.6. Application of the Periodic DFT Calculations in the Drug Analysis

Probably one of the most common applications of the periodic DFT calculations is the generation of spectral properties, such as NMR chemical shifts or IR/Raman signal frequencies and intensities in order to either confront them with the experimental data or to model them when an experiment is not possible. Such support of the analytical results with DFT calculations helps to explain the obtained experimental results, to eliminate the possible presence of an impurity, to validate the calculation method and optimize the structural parameters. A relatively common practice is to use DFT-based calculations to obtain the thorough information on the structural, electronic and absorption properties of a system (including generation of spectra), lately, often for the high-pressure polymorphs [164,165]. Optical absorption is also applied, on the basis of band gap data, to describe the potential insulator character of a molecule, as in the case of anhydrous haloperidol crystal [166].

Application of periodic DFT to obtain various spectroscopic data for one structure shows the wide range of DFT calculations’ possibilities [167]. It helps to successfully explain some atypical mechanisms like unique α resorcinol → β resorcinol transition where the high-temperature phase is a denser one (combination of theoretical INS and Raman) [168] or to solve properly the peak assignment which, based solely on the experimental data, has been under debate for years (theoretical IR, INS, NMR) [169].

#### 2.6.1. Vibrational Properties

Calculated through DFPT, the phonon density of states not only enables to obtain a precise description of a thermodynamic state [161] by calculating the Gibbs free energy, enthalpy, entropy, or zero-point energy but also the generation of vibrational spectra such as IR, Raman, and INS. All these calculations are usually characterized by a very good agreement with the experimental results [170,171] and in many cases they reveal the reason (e.g., molecular structure in itself, crystal packing) for spectral differences between two polymorphs [172].

However, some problems must be indicated. In the case of INS, the Perdew-Burke-Ernzerhof (PBE)-calculation has been repeatably reported to underestimate of the C=O stretching mode [173]. For IR spectra, the most discrepancies between calculation and experiment are always found in the far infrared region (low wave numbers) [174,175]. However, it is definitely less common when periodic DFT and not a calculation on a single molecule is performed. This is due to the insufficient description of the long-range Coulombic forces and charge polarization in the non-periodic approach [176].

Here, a slightly more detailed explanation of an attempt to calculate the vibrational properties is needed in order to fully feature the development of periodic DFT calculations and to stress the advantage of implementing them into the vibrational research. Generally speaking, there are a couple of factors which for a long time have prevented the theoretical science to properly foresee the low frequency vibrational spectra. Firstly, the sole nature of low frequencies area (so-called molecule fingerprint) which strongly depends on the intermolecular interactions [177]. Thus, the very accurate calculation method of such interactions must be implemented. Secondly, the DFT calculations are often performed for 0 K which neglects temperature contribution. Thirdly, the vibrational dynamics is often conducted within the harmonic approximation what naturally means neglection of any anharmonicity like temperature dependence [178]. This is why, for an accurate description of vibrational properties, a quasi-harmonic approximation [179] must be applied. As a result, determination of the temperature influence on the structural properties as well as description of volume-dependent thermal features is possible. Many results confirm that such an approach distinctly enhances the quality of the obtained vibrational spectra [180,181].

#### 2.6.2. Solid state NMR

**Figure 6:**
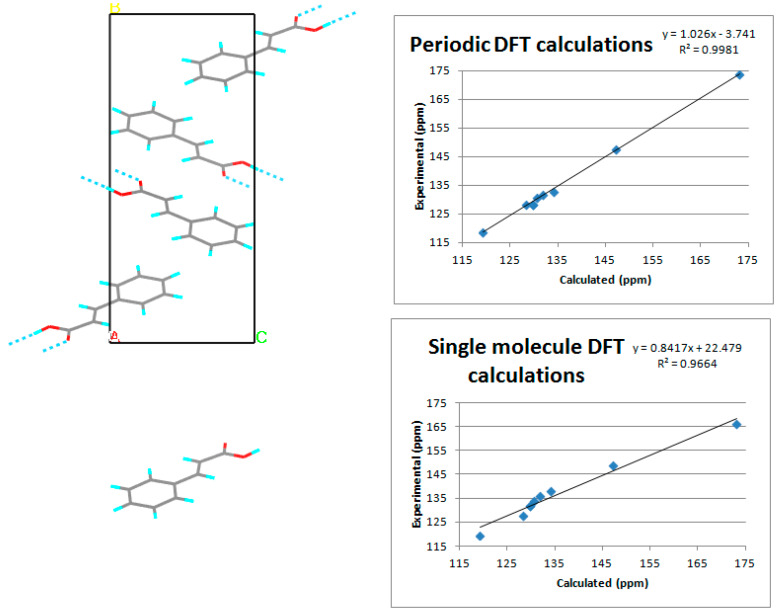
Results of Nuclear Magnetic Resonance (NMR) calculations for trans-cinnamic acid. On top using periodic DFT (GIPAW CASTEP), below using single molecule (GIAO Gaussian). By using periodic DFT calculations not only higher coefficient of determination (R^2^) but also slope closer to 1 were achieved. Source: author’s archive, more details [187].

Unambiguous signal assignment in solid state NMR is usually more challenging than in liquid state NMR because of the lack of the easy to obtain 2D spectra. That is why the DFT calculation are of great assistance in performing the spectral assignment. More information on the implementation of NMR methods in solid-state codes can be found in those reviews [182,183] and original theory papers [184,185,186]. Application of GIPAW-generated solid state nuclear magnetic resonance (ssNMR) spectra in order to assign the peaks is a common technique and its dominance over chemical shifts calculation on the single molecule or a cluster (Gauge Invariant Atomic Orbitals (GIAO)), is already known for at least a decade [187] (Figure 6). Most commonly NMR parameters calculations are performed for ^1^H and ^13^C or ^15^N [188] and ^35^Cl nuclei [189] but this methodology gives a reasonable result for many other isotopes like: ^23^Na [190] or ^31^P [191]. Numerous studies have already reported an excellent agreement of obtained spectra with the experimental results [192,193,194]. Calculation-derived spectra help to clarify the conflicting assignment of peaks [195] and often play a decisive role when only coarse PXRD data are available [196]. Already, a term, “NMR crystallography”, has been created [197,198,199] to describe an approach which combines performing experimental NMR and generating spectra with a DFT-based software in order to confirm or create the crystal structure of the studied system. It has been already proven that a research constructed in this way delivers more accurate data, compared to the separate application of these two techniques [200,201,202]. In some cases it is plainly stated that the proper designation of the hydrogen positions would not be possible at all, unless the combination of DFT and ssNMR had been used [203].

DFT-NMR helps to accurately define the moment of a phase transition and, in combination with experimental techniques, ends up with a straightforward identification of a substance. This is especially important when obtaining a pure API is very costly [204]. Often it is easier to first analyze (both experimentally and theoretically) the excipients [205] and afterwards put under close investigation the overall results with the generated API spectra.

NMR signals derived from the DFT calculations are also effectively applied in the pre-experimental phase of a research. For example, predicted via calculation of NMR shielding results for simple magnesium compounds served as a calibration for further analysis of more complex magnesium phases [206].

**Figure 7:**
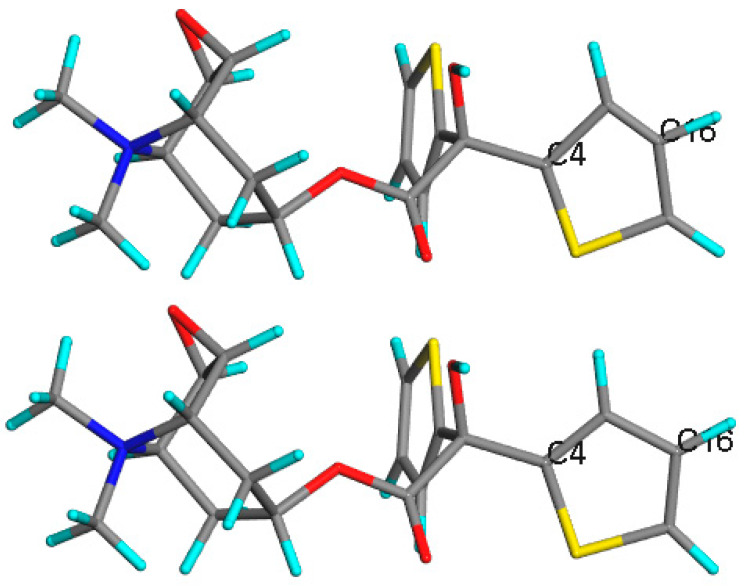
Results of GIPAW NMR calculations for tiotropium bromide monohydrate. On top is the experimental structure with solely hydrogen atoms positions optimized (188.54 ppm for C6 and 107.01 ppm for C12), below is the structure after all atom’s positions optimization (151.24 ppm for C6 and 127.64 ppm for C12). Experimental values: 148.91 ppm for C6 and 127.77 ppm for C12. Source: author’s archive, more details [207].

Theoretically generated NMR also plays a verifying role. In the case of tiotropium bromide (Figure 7) it helped us to correct the major errors in the already solved and widely accessible crystal structure [207]. At first, the Cambridge Crystallographic Data Centre (CCDC) structures have been used as a starting point for the NMR shielding constants calculation. Comparison of the obtained data with the experimental ssNMR results showed the incompatibility. Taking into account the fact that the GIPAW calculations are very sensitive to the atom positions, CSD structures have been put under closer investigation and the errors have been detected. Afterwards, the DFT calculations have been applied to optimize the structure of tiotropium bromide. The resulting from the calculations new structural data has been confirmed by the comparison with ssNMR and PXRD experimental and theoretical results.

The accuracy of combined ssNMR and DFT NMR (GIPAW calculation) is already confirmed so well that such an approach is used as a verifying tool for other techniques, like X-ray photoelectron spectroscopy (XPS). XPS has been used to establish that a new complex theophylline-5-sulfosalicylic acid dihydrate is a salt [208]. The fact of protonation has been proven by experimental and theoretical NMR, thus legitimizing XPS as an accurate method to determine a proton transfer in acid-base complexes.

The above described examples show that there exists a wide spectrum of possible usage of the DFT-generated NMR data. In fact, the assignment of peaks with the support of this technique has been already described and verified in numerous works [209,210,211,212,213,214].

## 3. Conclusions

The large amount of presented cases of applications of periodic DFT calculations in pharmaceutical sciences not only proves their versatility but also highlights their increasing importance. Further, it has been shown that the DFT level of theory provides the high accuracy of computational results as well as enables the calculations on real size systems in a reasonable time. A large variety of presented studies confirms that DFT periodic calculations for pharmaceutical systems can be successfully used to answer the fundamental questions as well as to provide the specific solutions for experimental challenges. The topics mentioned in this review included validation of the experimentally obtained crystal structures and crystal structure prediction, insight into crystallization and solvation processes, development of new polymorph synthesis ways and formulation techniques, as well as application of the periodic DFT calculations in the drug analysis using solid state analytical techniques such as Fourier Transform Infrared Spectroscopy (FT-IR) and NMR spectroscopy.

## Figures and Tables

**Table 1:** Selected software enabling periodic Denisty Functional Theory (DFT) calculations, most commonly applied in pharmaceutical sciences.

Software	Basis Set	Properties	Thermodynamic Properties	Calculation on a Single Molecule	License Type
CASTEP [27]	PW	+ ^a,b,c,d^	free energy, enthalpy, entropy, heat capacity, Debye temperature	+ *	Academic, commercial
DMol3 [28]	NAO	+ ^b^	free energy, enthalpy, entropy, heat capacity, heats of formation,	+	Commercial
DFTB+ [29]	NAO, STO	+ ^a,b^	free energy, enthalpy, entropy, heat capacity, heats of formation	+ *	Free, Lesser General Public License (LGPL)
Quantum Espresso [30]	PW	+ ^a,b,c,d^	free energy, enthalpy, entropy, heat capacity, Debye temperature	+ *	Free, General Public License (GPL)
CRYSTAL [31]	GTO	+ ^a,b,c^	free energy, enthalpy, entropy, heat capacity, Debye temperature	+	Academic, commercial
CPMD (Car-Parrinello Molecular Dynamics) [32]	PW	+ ^a,b,d^	free energy, enthalpy, entropy	+ *	Academic
SIESTA [33]	NAO	+ ^a,b^	free energy, enthalpy, entropy	+ *	Free, General Public License (GPL)
VASP [34]	PW	+ ^a,b,d^	free energy, enthalpy, entropy, heat capacity	+ *	Academic, commercial
CP2K [35]	GTO, PW	+ ^a,b^	free energy, enthalpy, entropy	+	Free, General Public License (GPL)

Basis set-type of basis set functions: PW–plane waves, GTO–Gaussian-type orbitals, NAO–natural atom orbitals, STO–Slater-type orbitals. Properties: list of properties that can be calculated (IR ^a^, Raman ^b^, INS ^c^, NMR ^d^) Phonon calculations: the possibility of lattice vibrations calculations and the supported formalism, Thermodynamic properties—thermodynamic quantities that can be calculated, Calculation on a single molecule—“+” means that both periodic and nonperiodic calculations are possible. “+ *” means that in order to perform the calculations on single molecules “molecules in the box” approach must be used, as described in the introduction.
